# Late-onset rheumatoid arthritis: clinical features, diagnostic challenges, and treatment approaches

**DOI:** 10.1007/s00296-025-05908-1

**Published:** 2025-06-09

**Authors:** Olena Zimba, Chokan Baimukhamedov, Burhan Fatih Kocyigit

**Affiliations:** 1 Department of Rheumatology, Immunology and Internal Medicine, University Hospital in Krakow, Krakow, Poland; 2 National Institute of Geriatrics, Rheumatology and Rehabilitation, Warsaw, Poland; 3 Department of Internal Medicine N2, Danylo Halytsky Lviv National Medical University, Lviv, Ukraine; 4 South Kazakhstan Medical Academy, Shymkent, Kazakhstan; 5 Shymkent Medical Centre of Joint Diseases, Shymkent, Kazakhstan; 6 Department of Physical Medicine and Rehabilitation, University of Health Sciences, Adana City Research and Training Hospital, Adana, Türkiye

**Keywords:** Rheumatoid arthritis, Late-onset rheumatoid arthritis, Geriatrics, Aged, Comorbidity

## Abstract

Late-Onset Rheumatoid Arthritis (LORA) is receiving increased clinical attention due to global aging trends. LORA presents distinct diagnostic, clinical, and therapeutic challenges. It often presents with a balanced gender distribution, acute onset, preferential involvement of larger joints, and decreased seropositivity. The diagnostic process is complex due to atypical presentations, comorbidities, and limitations of classification criteria, which insufficiently address the heterogeneity of LORA phenotypes. Patients with LORA often experience age-related geriatric syndromes, including frailty, cognitive decline, and malnutrition, in addition to comorbid cardiovascular disorders, pulmonary involvement, oncologic conditions, and osteoporosis. All these factors confound disease progression and treatment strategies, necessitating careful consideration of polypharmacy and modified drug metabolism. While the treatment principles largely align with those of Younge-Onset Rheumatoid Arthiritis (YORA), LORA management requires individualized approaches. Available evidence suggests that with proper monitoring, disease-modifying anti-rheumatic drugs (DMARDs) are safe and effective for older adults. Glucocorticoids should be minimized due to potential detrimental effects. Despite elevated baseline disease activity and functional deterioration, effectively managed LORA patients may achieve disease control similar to that of younger individuals. This review advocates for age-adjusted diagnostic strategies and patient-centered care models tailored to the needs of older RA patients. Addressing these unmet needs may enhance outcomes and quality of life for the growing population of LORA patients.

## Introduction

Rheumatoid arthritis (RA) is a progerssive systemic autoimmune disease characterized by persistent synovitis, systemic inflammation, and joint degeneration, resulting in disability and increased mortality [1, 2]. The traditional definition of Late-Onset Rheumatoid Arthritis (LORA) refers to the manifestation of RA after the age of 60 [3]. However, the age limit for managing LORA is still debated since no expert consensus is available [4].

The global increase in life expectancy, coupled with the continued demographic transition toward aging populations, signify age-related chronic diseases as public health priorities [5]. Progress in healthcare and living conditions has contributed to enhanced longevity of subjects aged above 60 [6]. In this regard, LORA is becoming a more prominent entity with distinct diagnostic and treatment issues [7]

A key issue in the field is the ambiguity of terminology. Terms such as “elderly,” “aged,” or “old age” are used inconsistently and may carry sociolinguistic or cultural biases [8]. Numerous research reports and practice guidelines refer to varying age thresholds for defining LORA (60 and 65 years) [9–12]. The term LORA per se is preferarable since it allows avoiding ageist approaches.

LORA clinically differs from Younge-Onset RA (YORA) in several key aspects. It is often less seropositive, manifest more acutely, and coexists with comorbidities such as osteoarthritis, cardiovascular disorders, osteoporosis, and neoplasms [9, 13, 14]. All these factors complicate and delay RA diagnosis and management. The management of LORA is particularly complex due to immunosenescence, multimorbidity, and polypharmacy. Older individuals often exhibit altered pharmacokinetic and pharmacodynamic responses, which increase their susceptibility to adverse effects [15]. Customized dose adjustments and improved safety monitoring are often essential, especially in subjects with kidney issues, liver impairment, or frailty [16]. Despite these challenges, current practice guidelines provide limited points on LORA management, mainly relying on evidence from YORA studies.

## Aim

This review aims to overview LORA as a unique clinical phenotype of RA. It seeks to clarify the distinct clinical, serological, and radiological features of LORA compared to YORA, considering diagnostic difficulties, comorbidity challenges, and treatment complexities associated with aging. The review also attempts to explore the limitations of current classification criteria, justifying the need for age-adjusted strategies.

### **Search strategy**

Comprehensive searches through Medline/PubMed, Scopus, Web of Science, and the Directory of Open Access Journals (DOAJ) were performed in April 2025 in line with previously published recommendations [17]. The following search term combinations were employed: ‘Late Onset Disorders and Rheumatoid Arthritis’ or ‘Elderly and Rheumatoid Arthritis’ or ‘Geriatrics and Rheumatoid Arthritis’ or ‘Aged and Rheumatoid Arthritis.’ No timeline filters were set for bibliographic searches. Analyses included English documents only.

### **Clinical characteristics of LORA**

#### Sex distribution

YORA predominantly affects women. In contrast, LORA shows a more balanced sex distribution, nearing a 1:1 ratio [18]. Available evidence points to a notably higher percentage of males in LORA cohorts compared with YORA [14, 19]. This shift in the sex ratio may suggests the existence of diverse disease triggers in elderly and younger individuals. One plausible explanation is that age-specific hormonal shifts, which significantly affect younger women, may diminish after menopause, thereby reducing female predisposition in later life [20].

#### Acute versus chronic onset

YORA progresses with fluctuations. Its symptoms evolve gradually in the context of moderately progressive course. LORA is characterized by a sudden and rapid development of symptoms, often resembling an infection. LORA patients may suffer from a rapid intensification of symptoms, including severe joint pain, edema, and fatigue [21]. Identifying these variations in disease onset is essential for timely diagnostic procedures and personalized therapeutic strategies [22].

#### Joint involvement

Joint involvement in LORA illustrates similarities and distinctive features in relation to YORA. Although the symmetrical rheumatoid polyarthritis is present in most older-age cases, pattern, intensity, and distribution of joint involvement in LORA vary considerably. LORA patients often present with involvement of large joints (e.g., shoulders, knees, hips), especially during the initial stages of the disease [23]. Shoulder Involvement is significantly more prominent in LORA and may resemble other diseases, such as polymyalgia rheumatica, thereby complicating differential diagnosis. In some older-age patients, oligoarticular or polymyalgia-like presentations may precede overt manifestations of RA, particularly in instances of seronegativity. Additionally, LORA more frequently involves proximal joints [14].

#### Clinical forms

LORA may present with three clinical forms. The most frequent form (75%) is consistent with typical RA, which is distinguished by rheumatoid factor (RF) positivity and progressive joint destruction [18].

The second clinical form (25%) is reminiscent of polymyalgia rheumatica, encompassing proximal limb joints [24]. It is predominantly seronegative, without erosions, and with acute presentation and beneficial prognosis [25].

The third form shares features with remitting seronegative symmetrical synovitis with pitting edema (RS3PE), a syndrome characterized by sudden onset of polyarthritis, pitting edema in the hands, male predominance, higher acute-phase reactants, and favorable prognosis [26].

Advancing bone deterioration is more characteristic for LORA [27]. One trial found that the enhancement in DAS28-ESR after a year was similar for both LORA and YORA; however, the residual synovial thickness and power Doppler signals assessed through ultrasound, along with radiographic progression, were considerably poorer in LORA [14]. Additional evidence suggests that radiological progression is more pronounced in LORA [28]. Nonetheless, the idea that LORA is inherently more progressive than YORA is not widely supported [29]. A Japanese registry study involving more than 1700 patients aged above 60 found no notable differences in treatment responses and functional outcomes between those with late and younge RA onset [30].

#### Laboratory characteristics

Serological characteristics of LORA differ substantially from YORA, indicating a more diverse and diagnostically challenging range.

#### Seropositivity patterns

Recent studies have highlighted the influence of aging on serological markers in RA, particularly RF and Anti-Citrullinated Peptide Antibody (ACPA) levels [31]. A large cohort study of 1685 RA patients revealed a gradual, age-related decline in both RF and ACPA positivity [13]. Specifically, the positivity rates for both antibodies showed a nearly linear decline with aging starting from 30 years, with RF positivity dropping from 80% in patients in their 30s to 51% in those aged above 80, while ACPA positivity decreased from 80% to 37% in the same age range.

The ANSWER cohort study revealed that RF positivity was considerably lower in LORA patients than in YORA (74% vs. 82%), whereas ACPA positivity revealed no significant difference between the two groups (84% vs. 85%) [19].

In the KURAMA cohort, patients with LORA showed lower positivity for both RF and ACPA than the same in YORA, though these differences lacked statistical significance [28]. Notably, within the ACPA-positive group, ACPA titers were substantially higher in LORA patients than in those with YORA [28]. While ACPA positivity may slightly decrease with age, higher titers in older patients could be linked to a more severe disease course, evidenced by increased rates of baseline and 2-year bone erosions observed in ACPA-positive LORA patients.

Taken together, these findings highlight the diversity of seropositivity features in LORA owing to diverse populations examined and variable study methods employed. Among seropositive LORA patients, heightened ACPA titers may indicate a more severe disease course. Subsequently, health professionals should cautiously interpret serological profiles in older individuals, taking into account both presence and titres of autoantibodies.

#### Inflammatory markers

LORA is often presents with elevated systemic inflammatory markers, exceeding the levels observed in YORA [32]. A Polish observational study revealed significantly raised C-Reactive Protein (CRP) and Erythrocyte Sedimentation Rate (ESR) in LORA patients, correlating with disease activity and remission rates [33]. Ke et al. [23] noted a median CRP level of 36.1 mg/L in LORA patients compared to 18.2 mg/L in YORA, underscoring LORA’s higher inflammatory disease profile.

Red cell Distribution Width (RDW) has also been identified as a measure of chronic inflammation. In the study by Ke et al., RDW was independently associated with both physical limitations and radiographic progression in older RA patients [23]. Furthermore, ferritin and D-dimer concentrations were significantly elevated in LORA patients, indicating a more intense or prolonged inflammatory response.

#### Geriatric syndromes and major comorbidities

LORA manifests with age-related physiological shifts, geriatric syndromes, and multimorbidity. Managing LORA requires a thorough comprehension of both inflammatory joint disease and complex age-related shifts.

#### Geriatric syndromes in LORA

Geriatric syndromes are prevalent among LORA patients. These include cognitive impairment, depression, falls, malnutrition, urinary incontinence, sarcopenia, and frailty [34, 35]. These syndromes affect multiple organ systems, worsening functionality and prognosis [36].

Chen et al. [37] reported that more than 55% of older RA patients presented with at least one geriatric syndrome, with cognitive impairment and falls occurring significantly more often in LORA than in YORA. Importantly, geriatric syndromes were independently associated with rheumatoid activity and anemia [36].​.

Falls, cognitive impairment, reduced physical activity, and sarcopenia are associated with both age-related physiological decline and systemic rheumatoid inflammation [38]. These syndromes often remain under-recognized in routine rheumatologic care, despite their impact on physical function and treatment adherence [36, 39–41].

#### Comorbidities

LORA frequently presents with multiple comorbidities that affect the disease progression and treatment:

- **Cardiovascular Disease**: Corticosteroids and nonsteroidal anti-inflammatory drugs contribute to cardiovascular risk mechanisms linking inflammation and atherosclerosis in rheumatic disorders, particularly in LORA [33]. Metabolic parameters, such as body mass index, serum creatinine, and uric acid, are more elevated in patients with LORA compared to those with YORA [33, 42].
- **Pulmonary involvement**: Pulmonary comorbidities frequently add to morbidity and mortality in LORA. Older age, male gender, and elevated disease severity are established risk factors of LORA [43]. From a geriatric perspective, deteriorating lung function, accumulated environmental exposures (e.g., smoking), and polypharmacy exacerbate respiratory conditions [44].
- **Malignancies**: Malignancy is yet another serious comorbidity in RA, particularly in LORA. RA per se is an independent risk factor for lymphoma, particularly in subjects with high rheumatoid activity and RF positivity [45]. In older RA patients, the importance of cancer screening and ongoing monitoring should be essential components of comprehensive RA management [46, 47].
- **Osteoporosis**: Osteoporosis in LORA is influenced by systemic inflammation, pharmacologic treatments, and reduced mobility. Chronic inflammation in RA promotes bone resorption through pro-inflammatory cytokines, which stimulate osteoclastogenesis and inhibit osteoblast function, thereby accelerating bone loss [48]. Furthermore, prolonged corticosteroid therapy, frequently employed in LORA, has been associated with significant reductions in bone mineral density [49, 50].
- **Infections**: Infections in LORA are confounded by immunosenescence, high rheumatoid activity, and treatment-induced immunosuppression [51]. A higher baseline inflammatory burden in LORA, linked to disability and immobility, increases the risk of respiratory and urogenital infections [52]. Diminished pulmonary capacity and age-related changes in genitourinary function further heighten this vulnerability in older adults [21].

### **Limitations of the 2010 RA classification criteria and the need for age-adjusted diagnostic strategies**

The 2010 ACR/EULAR classification criteria for RA were developed to facilitate early diagnosis and treatment, focusing on patients with early-stage disease [53]. While these criteria have improved sensitivity among younger populations [54], their applicability to LORA remains challenging. LORA patients often present with atypical features, including reduced involvement of small joints, seronegativity, and comorbidities such as osteoarthritis and polymyalgia rheumatica [14, 23]. These factors limit their capacity to meet the required classification criteria.

The main limitation of the classification criteria relates to the emphasis on joint engagement and serologic indicators within the scoring system. LORA patients often exhibit oligoarticular and large joint involvements and RF negativity, resulting in diagnostic delays and misdiagnoses [55].

In light of these issues, there is an urgent need for LORA-specific criteria that distinguish its features. Comprehensive international multicenter cohort studies are necessary to establish more precise classification criteria for LORA [56, 57].

### **Treatment**

**Fig. 1:**
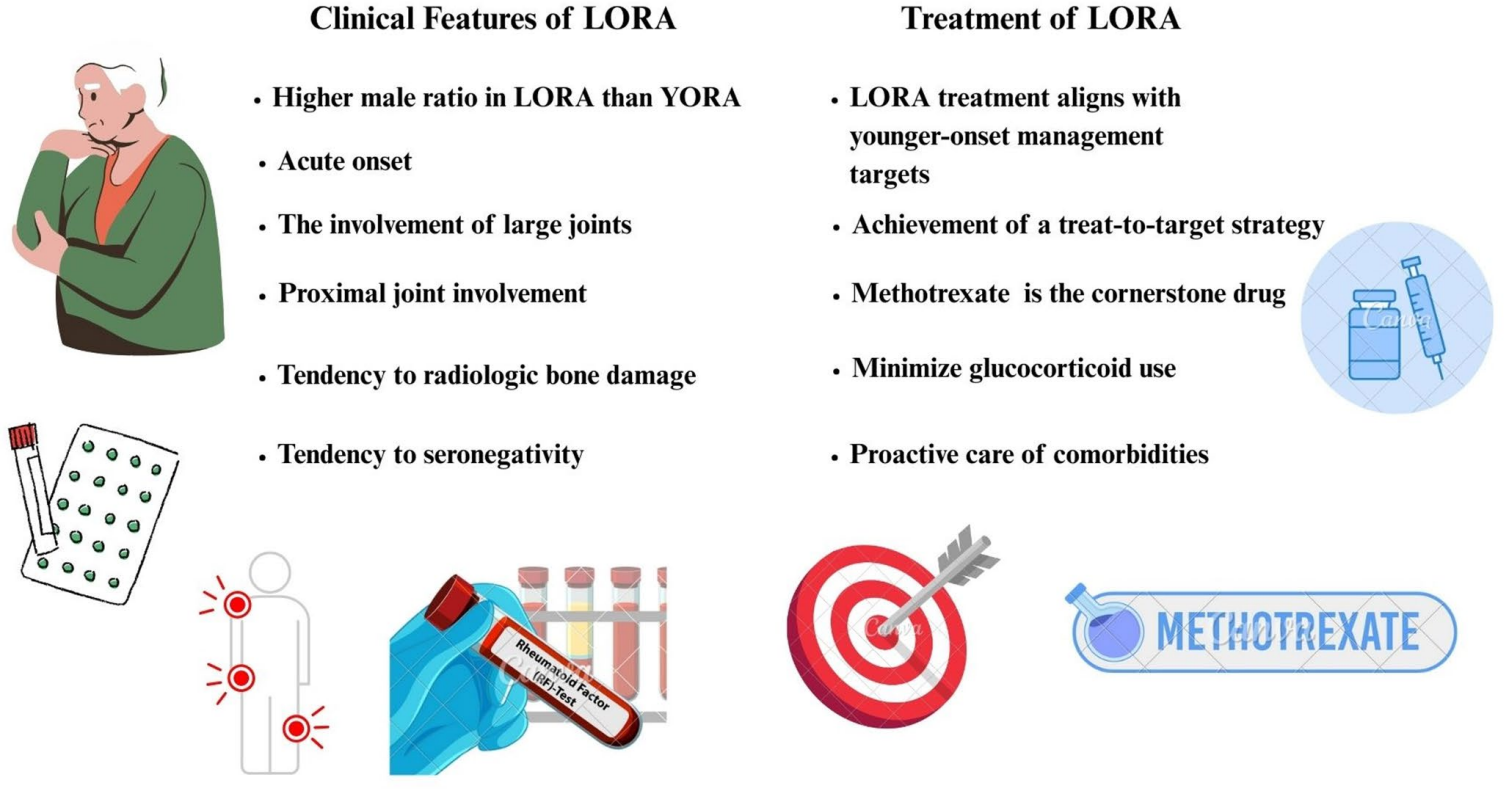
Clinical features and treatment of LORA

The aims of treatment strategies in LORA and YORA are the same (Fig. 1). In fact, the management of LORA should not differ from that of YORA [18]. Relevant EULAR recommendations emphasize that treatment strategies should be based on disease activity and take into account comorbidities and safety considerations [58].

While the core treatment approaches for LORA are similar to those for YORA, managing older adults involves distinct challenges stemming from age-related physiological alterations, multiple comorbidities, and adverse drug reactions. Complexities of polypharmacy, drug interactions toxicity, and adherence are evident in patients experiencing cognitive decline and lacking adequate caregiver support [59, 60].

A Japanese consensus statement on LORA states that methotrexate (MTX) is the cornerstone medication, with the necessity to escalate biologic or targeted synthetic DMARDs when treatment objectives are not achieved [61]. A treat-to-target (T2T) strategy can also be applied to older RA patients as long as comorbidities are properly managed [62]. Due to age-related decline in renal function, MTX may lead to increased toxicity in elderly patients. In such cases, leflunomide may serve as a suitable alternative, given its safety profile and tolerability in older individuals, including those with chronic kidney disease [63, 64].

The prolonged use of glucocorticoids in LORA has been challenging due to risks of adverse effects [65]. The GLORIA trial provided valuable evidence in this regard [66]. In this double-blind, placebo-controlled study, RA patients aged above 65 years received low-dose prednisolone (5 mg/day) for two years. The findings indicated that this treatment approach markedly diminished disease activity compared to the placebo group. Notably, despite a non-negligible rise in adverse events—predominantly mild to moderate infections—the overall assessment of benefit versus harm was favorable. A systematic review revealed that older individuals using biologic drugs experienced reduced efficacy and increased risk of adverse events compared to younger patients [67]. Older RA patients demonstrated more severe initial disease activity and longer disease duration, which could contribute to worse outcomes. A retrospective multicenter study on tocilizumab involving a cohort of RA patients, slightly more than one-quarter of whom were 65 or older [68]. After six months of treatment, older patients exhibited significantly lower remission rates than younger patients, even after accounting for confounding variables (27.8% versus 45.6%; *p* = 0.02). Despite the observed reduction in efficacy, drug retention and discontinuation rates were comparable across the different age groups, suggesting that tocilizumab is well tolerated in older adults. An analysis conducted by the FIRST registry evaluated the efficacy and safety of b/tsDMARDs in older patients, categorizing them into LORA and non-LORA groups [30]. The study demonstrated that Clinical Disease Activity Index response trajectories, treatment retention rates, and adverse event profiles were comparable between the two cohorts over a duration of 54 weeks. These data suggest that b/tsDMARDs can be effectively and safely employed in LORA patients. The GISEA registry study involving RA patients aged 65 and older demonstrated that the primary reason for the discontinuation of b/tsDMARDs was a loss of efficacy, with comorbidities and high disease activity identified as significant risk factors [69]. Among the biologics, abatacept exhibited the highest retention rate, thereby indicating its potential as a favorable treatment option for elderly patients. These findings underscore that advanced age should not be regarded as a contraindication for the use of b/tsDMARDs, thereby advocating for individualized strategies that prioritize disease control.

The ANSWER cohort study compared the efficacy of bDMARDs in LORA and YORA [19]. Following adjustments for baseline discrepancies, clinical outcomes at 48-week point, including improvements in disease activity, remission rates, drug retention, and discontinuation due to adverse events, were similar in both cohorts.

Management of LORA requires a tailored and multifaceted approach due to challenges posed by aging, comorbidities, and heightened risk of adverse events. Studies indicate that, if properly monitored, b/tsDMARDs can be effective and safe in older RA patients [70, 71]. Achieving the best results in LORA requires balancing effective disease management with proactive care of comorbidities, reducing glucocorticoid use, and tackling geriatric-specific issues like polypharmacy, frailty, and cognitive decline. Ultimately, a patient-centered approach—founded on diligent monitoring and personalized care—is essential for enhancing both clinical results and quality of life in the expanding group of older patients.

One significant diagnostic challenge in LORA is differentiating it from other common conditions in older adults, especially polymyalgia rheumatica, crystal-induced arthropathies, and osteoarthritis. These conditions often involve larger joints and exhibit lower seropositivity rates, complicating the differentiation process. Misclassification can lead to the inappropriate use of DMARDs or corticosteroids, resulting in unnecessary adverse effects without clinical benefit. Consequently, clinicians should use a combination of serological, radiographic, and, where necessary, synovial fluid analysis to differentiate RA from other age-associated arthropathies [24, 32].

### Prognosis and long-term outcomes in LORA

The long-term outcomes of LORA are influenced by a mix of disease-related variables, age-associated medical conditions, and functional deterioration. In an Asian cohort study comparing LORA with YORA, both patient groups demonstrated similar disease activity levels and radiographic erosions at baseline [72]. LORA patients had a higher frequency of comorbidities [median 2 [inter-quartile range 1–3] vs. 1 (0–2), *p* < 0.001], reduced functional status [13.8% vs. 8.7%, *p* = 0.027], and underwent less intensive management with DMARDs [median one [IQR 1–2] vs. two (IQR 1–3), *p* < 0.001]. In the CATCH cohort trial, LORA patients exhibited higher baseline disease activity [*p* < 0.001 for DAS-28], impaired functioning [*p* < 0.001 for HAQ], and more instances of radiographic erosions [*p* < 0.001 for presence of erosions] than younger patients [73]. The older group received more cDMARDs [*p* < 0.001], steroids [*p* < 0.005], and fewer biologics [*p* < 0.001] and exhibited a comparable decrease in disease activity over 12 months [change in DAS-28, *p* = 0.261] relative to younger cohorts. Remission rates were markedly lower in the older-onset group. In the METEOR extensive international registry study, LORA patients exhibited distinct baseline characteristics, including higher inflammatory markers, more seronegative disease, and greater functional impairment than younger patients [74]. Despite these differences, treatment responses were similar across age groups, with no clinically significant differences in disease activity reduction or time-to-treatment escalation. Targońska-Stępniak [33] pointed out that even with comparable seropositivity and joint involvement, LORA leads to a greater inflammatory burden and poorer disease activity control, poor prognosis, and necessity for customized management approaches in older patients. A Swiss cohort study examined radiographic progression and disease activity in LORA compared to YORA patients during five years [75]. At baseline, LORA patients demonstrated greater disease activity, more joint erosions, and higher incidence of corticosteroid utilization, while being prescribed fewer cDMARDs and biologics. Both cohorts exhibited comparable radiographic progression and decreases in disease activity over time. The CORPUS cohort study compared RA treatment patterns and outcomes in patients above and below 75 years of age [76]. Older patients above 75 years were prescribed TNFα antagonists less often (6% vs. 32%, *p* < 0.01)] and relied more on glucocorticoids. Although inflammation markers and disability scores improved over one year in both groups, older patients had higher comorbidity burden, influencing treatment choices.

**Fig. 2:**
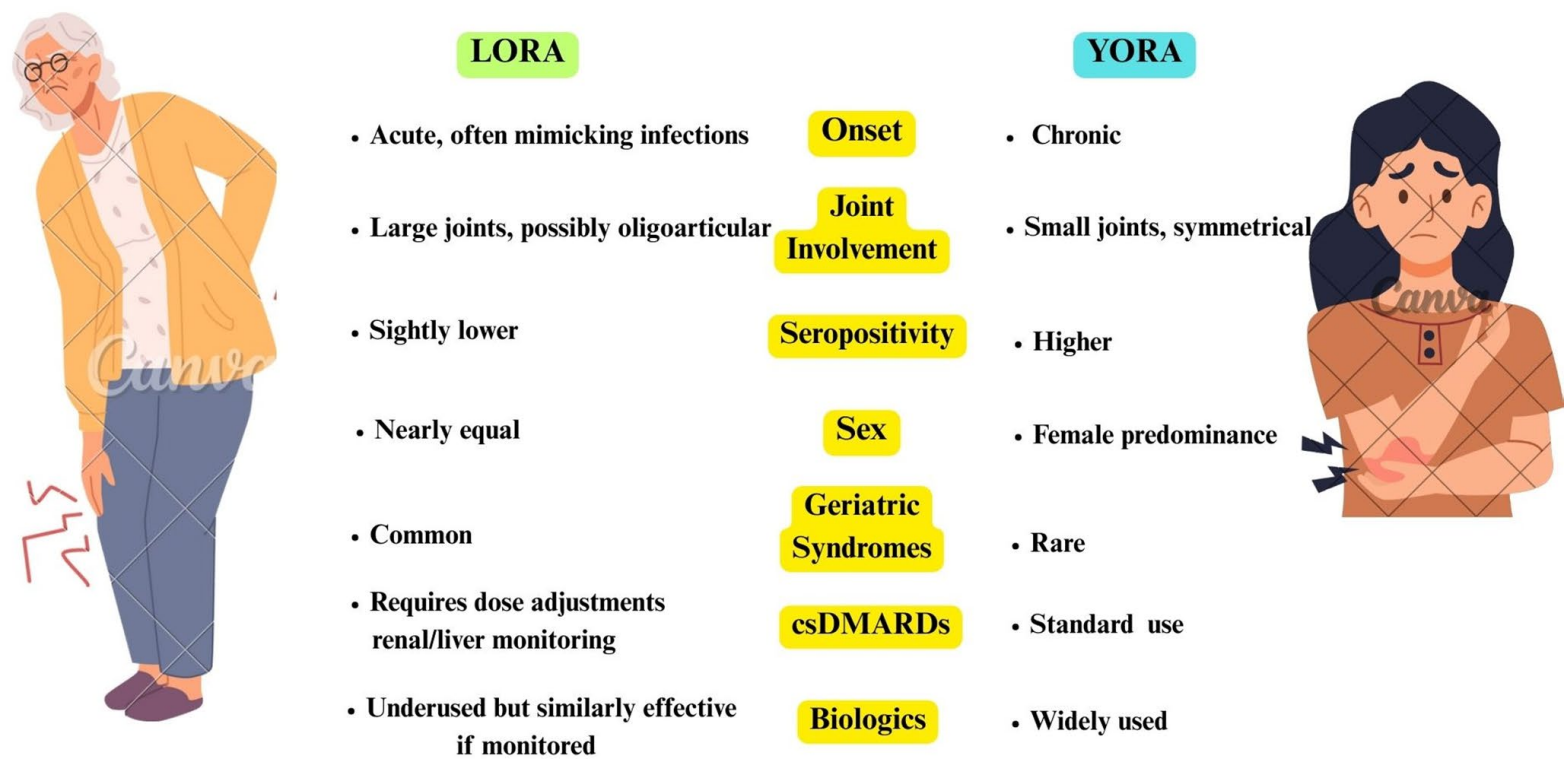
Comparative overview of LORA and YORA

To sum up, these findings underscore the heterogeneity and complexity of LORA (Fig. 2). Optimizing long-term outcomes in LORA requires a shift toward individualized, proactive management strategies prioritizing effective disease control without undue therapeutic restraint.

### Perspectives

Existing diagnostic and treatment paradigms, primarily based on younge RA groups, inadequately address the difficulties arising from age-related physiological alterations, multimorbidity, and geriatric disorders.

Future treatment approaches should emphasize individualized treatment, ensuring optimal disease management while minimizing adverse effects. This entails optimizing the administration of DMARDs and other medications in patients above 60 years, underpinned by comprehensive risk assessment instruments that incorporate comorbidity profiles, frailty indicators, and functional status. The appropriate and proactive application of glucocorticoids, guided by current trials, should be standardized in LORA-specific guidelines. DMARDs should be administered promptly to achieve early disease control and prevent structural damage. Regular clinical and laboratory monitoring is crucial for evaluating therapeutic response and minimizing potential adverse effects. Furthermore, strict adherence to established treatment guidelines is essential to avoid prolonged glucocorticoid dependency and ensure optimal, evidence-based care.

An essential strategic target is the integration of multidisciplinary management models in which rheumatologists cooperate closely with geriatricians, physical medicine and rehabilitation specialists, nutritionists, and mental health professionals. Rehabilitation initiatives, nutritional procedures, and preventive measures should be integrated into routine LORA management to maintain patients' functional independence and quality of life.

## Declarations

### Disclaimer

Authors state that no part of this manuscript, including the text and graphics, are copied or published elsewhere in whole or in part.

### Figures

Canva was used to create the Figures.
